# Patient Age, Race and Emergency Department Treatment Area Associated with “Topbox” Press Ganey Scores

**DOI:** 10.5811/westjem.2020.8.47277

**Published:** 2020-10-19

**Authors:** Moon O. Lee, Jonathan Altamirano, Luis C. Garcia, Michael A. Gisondi, N. Ewen Wang, Suzanne Lippert, Yvonne Maldonado, Laleh Gharahbaghian, Ryan Ribeira, Magali Fassiotto

**Affiliations:** * Stanford University School of Medicine, Department of Emergency Medicine, Stanford, California; † Stanford University School of Medicine, Office of Faculty Development and Diversity, Stanford, California; ‡ Kaiser-Permanente East Bay, Department of Emergency Medicine, Oakland, California

## Abstract

**Introduction:**

Hospitals commonly use Press Ganey (PG) patient satisfaction surveys for benchmarking physician performance. PG scores range from 1 to 5, with 5 being the highest, which is known as the “topbox” score. Our objective was to identify patient and physician factors associated with topbox PG scores in the emergency department (ED).

**Methods:**

We looked at PG surveys from January 2015–December 2017 at an academic, urban hospital with 78,000 ED visits each year. Outcomes were topbox scores for the questions: “Likelihood of your recommending our ED to others”; and “Courtesy of the doctor.” We analyzed topbox scores using generalized estimating equation models clustered by physician and adjusted for patient and physician factors. Patient factors included age, gender, race, ethnicity, and ED area where patient was seen. The ED has four areas based on patient acuity: emergent; urgent; vertical (urgent but able to sit in a recliner rather than a gurney); and fast track (non-urgent). Physician factors included age, gender, race, ethnicity, and number of years at current institution.

**Results:**

We analyzed a total of 3,038 surveys. For “Likelihood of your recommending our ED to others,” topbox scores were more likely with increasing patient age (odds ratio [OR] 1.07; 95% confidence interval [CI], 1.03–1.12); less likely among female compared to male patients (OR 0.81; 95% CI, 0.70–0.93); less likely among Asian compared to White patients (OR 0.71; 95% CI, 0.60–0.83); and less likely in the urgent (OR 0.71; 95% CI, 0.54–0.93) and vertical areas (OR 0.71; 95% CI 0.53–0.95) compared to fast track. For “Courtesy of the doctor,” topbox scores were more likely with increasing patient age (OR 1.1; CI, 1.06–1.14); less likely among Asian (OR 0.70; 95% CI, 0.58–0.84), Black (OR 0.66; 95% CI, 0.45–0.96), and Hispanic patients (OR 0.68; 95% CI, 0.55–0.83) compared to White patients; and less likely in urgent area (OR 0.69; 95% CI, 0.50–0.95) compared to fast track.

**Conclusion:**

Increasing patient age was associated with increased likelihood of topbox scores, while Asian patients, and urgent and vertical areas had decreased likelihood of topbox scores. We encourage hospitals that use PG topbox scores as financial incentives to understand the contribution of non-service factors to these scores.

## INTRODUCTION

In 2008, the Institute for Healthcare Improvement developed the Triple Aim framework to optimize health system performance by focusing on the following: improving the patient experience of care; improving the health of populations; and reducing the cost of healthcare.^1^,^2^ Patient experience is often measured by patient satisfaction. Patient satisfaction is positively associated with improved physician-patient communication, medication compliance, provider job satisfaction, reductions in malpractice claims, and hospital profitability.^3^–^8^ Hospitals have used financial incentives to link physicians’ professional and financial success to their patient satisfaction scores. Some surveys demonstrated that up to 43% of physicians have some portion of their financial compensation linked to patient satisfaction measures.^9^

Press Ganey Associates Inc. (South Bend, IN) first developed patient satisfaction surveys in 1985, and have become the industry standard for measuring patient experience in the outpatient setting.^10^–^15^ Hospitals typically distribute Press Ganey (PG) standardized surveys to a random sample of patients to solicit feedback regarding providers, staff, and clinical environments. PG uses a five-point Likert scale for patient responses. A score of 5, the most favorable, is known as the “topbox” score.^13^,^16^ Topbox scoring is the standard for customer satisfaction and consumer research.^17^

Despite widespread adoption of patient satisfaction measurement systems and associated incentives, concern was raised about the validity of these tools since current literature does not consistently demonstrate key predictors of higher or lower scores.^18^ Only a few studies have examined PG surveys specific to the emergency department (ED); some studies have found that ED PG scores are positively associated with employee satisfaction and retention, and negatively associated with ED crowding and wait times.^6^,^19^,^20^ There is evidence that acuity of a patient’s illness and the patient care setting affect PG scores. Critical, emergent patients were more likely to give higher scores than non-urgent patients.^21^ Bendensky et al showed the same physicians had higher “courtesy of the doctor” scores from the urgent care setting than in the ED.^11^

Gender also influences the perceptions, behavior, and communication of patients and their providers.^22^,^23^ Patients have different expectations from male and female physicians.^24^ The ED setting is unique in that patients have unscheduled visits and cannot choose their healthcare provider in the ED. The influence of patient or physician factors specific to ED PG scores has been limited to a few studies.^21^–^23^,^25^ We hypothesized that patient factors (age, gender, race, and/or ED area where patient was seen) and physician factors (age, gender, race, years at institution) influence topbox scores for two ED PG survey questions: “Likelihood to recommend ED,” and “Courtesy of the doctor.”

**Population Health Research Capsule**

What do we already know about this issue?

*Press Ganey scores are often used to benchmark physicians. The relationship between patient and physician factors with the highest (topbox) score is unclear*.

What was the research question?

Are patient and physician factors associated with topbox scores on Press Ganey surveys?

What was the major finding of the study?

*Patient factors were associated with topbox scores, but physician factors were not associated with topbox scores*.

How does this improve population health?

*Physicians and administrators will be informed about the contribution of non-service factors associated with Press Ganey topbox scores*.

## METHODS

### Study Design and Setting

This was an observational, population-based study at an urban, academic, tertiary care hospital. The hospital is a designated Level I adult and Level I pediatric trauma center and a comprehensive stroke center. The annual ED volume is approximately 78,000 visits a year. The ED has a separate pediatric ED and adult ED. The adult ED is divided into different care areas based on age and patient acuity: emergent; urgent; vertical (urgent but able to sit in a recliner rather than a gurney); and fast track (non-urgent). The emergent area is for adult patients 18 years and older who require acute resuscitations, require trauma assessments, or are otherwise clinically high-risk patients. The urgent and vertical areas were designed for patients who do not require emergent intervention or assessment. The fast-track area was designed for patients over six months old who are triaged as non-urgent with an estimated discharge within 90 minutes. Approximately, eight ED attendings worked only during the overnight shift. The overnight physicians only worked in the emergent and urgent areas since these two areas were the only open areas on the adult overnight shifts. All other general emergency physicians worked in the different areas of the adult ED.

### Study Population

We collected PG survey data from January 2015–December 2017 for adult patients (age ≥18 years) who were evaluated, treated, and discharged from the ED. All patients enrolled in the online patient portal received a PG survey after their ED visits. For patients without the online portal access, five unique patients per physician per month were randomly selected to receive a paper survey. If patients had multiple visits with several physicians within 21 days, only one visit was randomly chosen for evaluation. Patients did not receive a PG survey from the ED if they had received a PG survey from the hospital within one week of the ED visit. This study was approved by the Stanford University School of Medicine Institutional Review Board.

### Measurements

#### Patient Factors

Self-reported patient demographic information obtained from PG surveys included age, gender, race, and ethnicity. Patients age 18–29 were grouped into age less than 30 years due to the small sample size. Patient age greater than 30 years was divided into 10-year intervals. Race and ethnicity were categorized as White, Asian, Black/African-American, Hispanic, Native American or Alaskan Native, and Native Hawaiian or other Pacific Islander. Surveys that reported race as “other” or “more than two racial backgrounds” were excluded from data analysis given low sample size in each category. We also excluded from the analysis surveys that reported race as “unknown”. The ED area where patients were seen and treated was provided for each PG survey.

#### Physician Factors

Physician demographic data included age, gender, race, ethnicity, and years at the current institution. Age, race, and ethnicity data were categorized into the same groups as the patients.

### Outcome

Two PG questions that are often used to inform hospital-related incentives for physicians were chosen for the outcomes. The two primary outcomes were topbox scores for “Likelihood of your recommending our emergency department to others,” and “Courtesy of the doctor.”

### Statistical Analysis

We used chi-squared tests of independence (χ^2^ tests) to assess the associations between patient and physician factors and impact of ED area on “Likelihood of your recommending our ED to others” and “Courtesy of the doctor” PG scores. Two generalized estimating equation (GEE) models were performed, one using topbox “Likelihood of your recommending our ED to others” as the outcome variable and the other using “Courtesy of the doctor.” Models controlled for patient and physician factors, and the ED area where the patient was seen. We used GEE models to cluster surveys by physician, using an exchangeable correlation structure to account for possible correlations within survey responses for the same physician. Models were performed using surveys with complete patient and physician demographic information. A *P* value ≤0.05 was considered statistically significant for all tests, and 95% confidence intervals were reported. We performed analysis in SAS 9.4 (SAS Institute, Cary, NC).

## RESULTS

**Figure 1:**
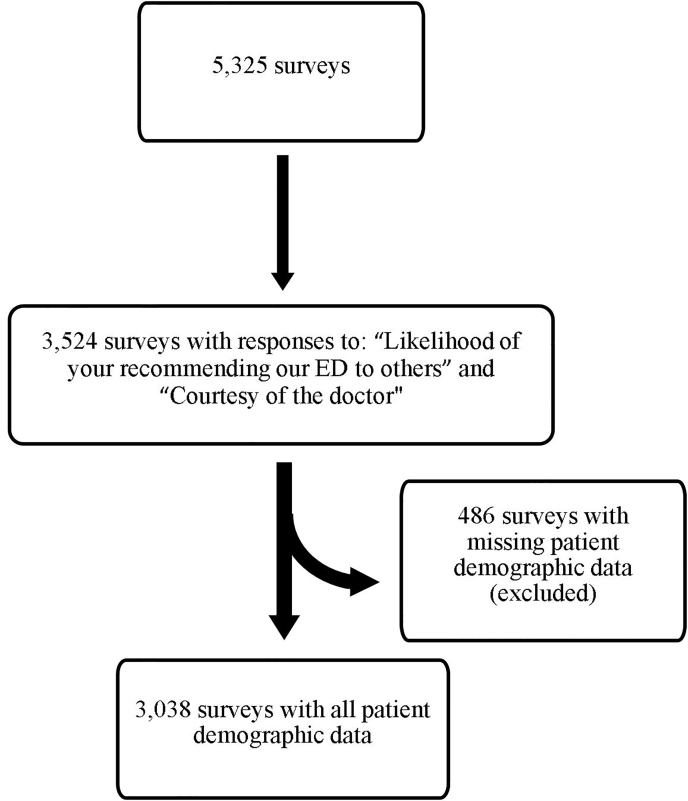
Study design of patients who completed Press Ganey surveys in the emergency department. *ED*, emergency department.

The response rate for ED PG surveys was 10%. Of the returned 5,325 surveys, 3,524 surveys answered both “Likelihood of your recommending our ED to others” and “Courtesy of the doctor” questions. From the 3,524 surveys with both outcomes questions answered, 3,038 surveys had complete patient demographic information including age, gender, race, and ethnicity. See Figure 1 for study design. Out of the 3,038 surveys, 2,400 were paper surveys, and 638 were online surveys. Most of the online responses 389 (61%) were in 2017. For each year of the study (2015–2017) the mean topbox scores “Likelihood of your recommending our ED to others” were 69%, 70%, and 66%. For each year, the mean topbox scores for “Courtesy of the doctor” were similar: 73%, 74%, and 72%.

### Patient Characteristics

Patients who responded to the PG survey did not mirror the demographics of the patients discharged from the ED. Women patients were 58% of the PG study population but only 53% of the ED discharge population. Patients over the age of 60 were 51% of PG study population, while patients over 60 years made up only 25% of the ED discharge population. White patients were 63% of the PG study population, but only 32% of the ED discharge population. Asian patients were 16% of the PG population and 14% in the ED discharge population. Hispanic patients were only 15% of the PG population, in contrast to 36% in the ED discharge population. Most patients were assigned to the urgent area (43%), and the next largest group was assigned to the vertical area (23%). Patient demographics are shown in Table 1.

### Physician Characteristics

Most of the PG surveys were completed for male physicians (64%). Physicians were younger than patients, with 76% of ED visits with physicians younger than 50 years old. Physician race was similar to that of the patient population, and most visits were with White physicians (75%). The mean number of years that a physician worked in the Stanford ED was eight years, standard deviation 9.1. Physician demographics are shown in Table 1.

### Chi-squared Tests Results

The proportion of topbox scores for “Likelihood of your recommending our ED to others” and “Courtesy of the doctor” by patient and physician gender, race, and ED area are summarized in Table 2. Female patients gave significantly fewer topbox scores than male patients for “Likelihood of your recommending our ED to others” and “Courtesy of the doctor” (*P* = 0.0023 and *P* = 0.027, respectively). Asian patients gave significantly fewer topbox scores than White patients for “Likelihood of your recommending our ED to others” and “Courtesy of the doctor” (*P* = 0.0018 and *P* <0.0001, respectively). Patients seen in urgent and vertical areas gave significantly lower topbox scores for “Likelihood of your recommending our ED to others” (*P* <0.0001) and “Courtesy of the doctor” (*P* =0.0008) than compared to fast track. Physician gender and physician race were not significantly associated with topbox scores for either question.

Chi-squared tests showed that gender concordance may influence “Likelihood of your recommending our ED to others” and “Courtesy of the doctor” (Table 3). After stratifying data by physician gender, female patients were shown to give significantly fewer topbox scores for “Likelihood of your recommending our ED to others” if the physician was also female (*P* = 0.01). Male patients did not show significant difference for topbox scores with physician gender.

### Generalized Estimating Equation Modeling Results for “Likelihood of Your Recommending Our ED to Others”

After controlling for patient and physician factors, we observed that patient age, patient gender and race, and ED area where they were seen were significantly associated with odds of a topbox score for “Likelihood of your recommending our ED to others” (Table 4). Each 10-year increase in patient age was associated with an increase in the odds of a topbox score (odds ratio [OR] = 1.07; 95% CI, 1.03 – 1.12, *P* = 0.001). Female patients had decreased odds of giving a topbox score when compared to male patients (OR = 0.81; 95% CI, 0.7 – 0.93, *P* = 0.003). Asian patients had lower odds of giving a topbox score when compared to White patients (OR = 0.71; 95% CI, 0.6 – 0.83, *P* <0.0001). Patients seen in the urgent area had lower odds of giving a topbox score when compared to patients seen in fast track (OR = 0.71; 95% CI, 0.54 – 0.93, *P* = 0.01), as did patients seen in the vertical area (OR = 0.71; 95% CI, 0.53 – 0.95, *P* = 0.02).

### Generalized Estimating Equation Modeling Results for “Courtesy of the Doctor “

After controlling for patient and physician factors, we observed that patient age, patient race, and ED zone were significantly associated with odds of receiving a topbox score (Table 4). Each 10-year increase in patient age was associated with increased odds of a topbox score (OR = 1.1; 95% CI, 1.06 – 1.14, *P* <0.0001). Asian (OR = 0.70; 95% CI, 0.58 – 0.84, *P* = 0.0001), Black (OR = 0.66; 95% CI, 0.45 – 0.96, *P* = 0.03), and Hispanic (OR = 0.68; 95% CI, 0.55 – 0.83, *P* = 0.0001) patients all had lower odds of giving a topbox score when compared to White patients. Patients seen in the urgent area had a significantly lower odds of giving a topbox score when compared to patients seen in fast track, (OR = 0.69; 95% CI, 0.50 – 0.95, *P* = 0.02).

## DISCUSSION

Our study found that patient factors were associated with topbox scores for PG questions while physician factors did not influence topbox scoring. As patients’ ages increased by decade, they were more likely to give topbox scores for “Likelihood of your recommending our ED to others” and “Courtesy of the doctor.” Asian patients and patients seen in the urgent and vertical zones of the ED were less likely to give topbox scores for “Likelihood to recommend emergency room” and “Courtesy of the doctor.”

Our study has multiple strengths that led to new results, which have not been previously published on PG surveys in the ED. First, our study detected a difference in race and topbox scores due to a diverse patient population. Boudreaux et al shows that ED patient demographics (age, gender, race) were unrelated to patient satisfaction scores but categorized patient race/ethnicity as “Black” or “other.”^25^ Due to our distinct patient population, we were able to demonstrate for the first time that Asian patients in the ED are less likely to give a topbox score compared to White patients. Second, our large study adds new information about patient satisfaction in the ED using topbox scoring. Topbox scoring is a more accurate measure for customer satisfaction in consumer research and is associated with predicting growth.^17^,^26^

A meta-analysis examined multiple PG studies in all specialties and found female physicians were slightly favored when the physician had less experience, when it was the first visit, and the survey was administered right after a visit.^27^ A subsequent study in 2017 by Chen et al found physician gender, ethnicity, and race were not associated with topbox scores, but the scores were associated with specialty; obstetrics and surgery had higher scores compared to medicine, but they did not examine emergency physicians.^28^ Milano et al examined PG surveys in the ED and in a small study of 398 surveys showed that the median score for “Courtesy of the doctor” of male emergency physicians and female emergency physicians did not significantly differ.^23^ Our study examined PG surveys over a three-year period with a large number of completed surveys, (n = 3,038) with topbox scores as our outcome.

A third strength of our study is that it is one of the few studies to have demonstrated significant association between the area of the ED where patients are seen and PG topbox scores. A prior study by Bendensky et al demonstrated that the mean score for “Courtesy of the doctor” was higher in the urgent care setting compared to the ED setting with the same physicians working in both locations.^11^ Boudreaux et al found “emergent” patients were more satisfied than “urgent” and “routine” patients with the ED visits. This study was based on the initial ED Emergency Services Index, which was determined at triage, and “routine” patients were seen in a rapid care area with a mean ED length of stay of 136 minutes.^21^ In contrast, our study demonstrated that patients seen in fast track were more likely to give topbox scores. In our fast track, patients were typically seen and discharged within 90 minutes of arrival to the ED. The second area of the ED associated with topbox scores was the “emergent” zone in which the most critical patients are seen, which is consistent with prior studies. Patients were least likely to give topbox scoring in the “vertical” zone where patients are classified as urgent, but able to sit in a recliner rather than a gurney.

Fourth, our study is the first to examine PG topbox scores in the ED and consider patient factors, physician factors, and the ED area where patients are seen. Prior studies of PG surveys in the ED focused only on physician gender and did not take into account patient gender or the area in the ED where the patient was seen.^23^ By accounting for all of these factors, we found that age of the patient, Asian patient race, and ED area were associated with topbox scores.

## LIMITATIONS

Our study has several limitations to consider. Our study is limited by the use of self-reported survey data that we cannot link with patient outcomes. Our response rate was 10%, which may have led to sampling bias. Patients who returned the survey may be different than those who did not respond. We did not have the response rate for each area of the ED, which may have led to sampling bias. Additionally, our study was conducted at one academic institution with a diverse patient population and may not be generalizable to other geographic areas of the country.

## CONCLUSION

Many hospitals use Press Ganey surveys as a measure of quality of care and provide financial incentives to physicians based on their scores. Our study demonstrates that patient race, patient age, and location where patients are seen in the ED are associated with PG topbox scores. We encourage hospitals that use PG topbox scores as financial incentives to understand the contribution of non-service factors to these scores.

## Figures and Tables

**Table 1:** Physicians’ and patients’ demographics for Press Ganey surveys.

Variable	Demographic data n = 3,038 n (%)
Survey year	
2015	798 (26)
2016	991 (33)
2017	1,249 (41)
Patient age, in decades	
18 – 30	274 (9)
31 – 39	381 (12)
40 – 49	320 (11)
50 – 59	512 (17)
60 – 69	548 (18)
70 – 79	571 (19)
80 – 89	334 (11)
90+	98 (3)
Patient gender	
Male	1,275 (42)
Female	1,763 (58)
Patient race/ethnicity	
White	1,907 (63)
Asian	489 (16)
Black	149 (5)
Hispanic	443 (15)
Native Hawaiian or Pacific Islander	43 (1)
American Indian or Alaskan Native	7 (0.2)
Emergency department zone	
Emergent	664 (22)
Urgent	1,312 (43)
Vertical	706 (23)
Fast Track	356 (12)
Physician age, in decades	
<30	13 (1)
31 – 39	1,170 (39)
40 – 49	1,100 (36)
50 – 59	367 (12)
60 – 69	388 (12)
Physician gender	
Male	1,956 (64)
Female	1,082 (36)
Physician race/ethnicity	
White	2,290 (75)
Asian	742 (24)
Black	6 (<1)

**Table 2:** Topbox, or highest scoring, surveys by physician and patient demographics.

	Topbox likelihood to recommend emergency department n = 3038		Topbox courtesy of the doctor n = 3038	

Variable	n (%)	P-value	n (%)	P-value
Patient gender				
Men	905 (71)		955 (75)	
Women	1,161 (66)	0.0023	1,259 (71)	0.027
Patient race and ethnicity				
White	1,338 (70)		1,452 (76)	
Asian	300 (61)		333 (68)	
Black	97 (66)		99 (67)	
Hispanic	293 (66)	0.0018	290 (65)	<0.0001
Emergency department zone				
Emergent	506 (76)		517 (78)	
Urgent	856 (65)		923 (70)	
Vertical	451 (64)		503 (71)	
Fast track	253 (71)	<0.0001	271 (76)	0.0008
Physician gender				
Male	1327 (68)		1,430 (73)	
Female	739 (68)	0.76	784 (72)	0.76
Physician race and ethnicity				
White	1,555 (68)		1,682 (73)	
Asian	509 (69)	0.69	531 (72)	0.38

**Table 3:** Topbox scores by patient and physician gender.

	Topbox likelihood to recommend emergency department		Topbox courtesy of the doctor	

Patient-physician gender	N (%)	P-value	%	P-value
Male physicians				
Male patients	589 (70)		626 (75)	
Female patients	738 (66)	0.06	802 (72)	0.22
Female physician				
Male patients	316 (73)		329 (76)	
Female patients	423 (65)	0.01	455 (70)	0.06

**Table 4:** Odds of “likelihood to recommend emergency department” and “courtesy of the doctor” topbox scores by physician and patient demographics.

	Likelihood to recommend emergency department		Courtesy of the doctor	

Variable	OR (95% CI)	P-value	OR (95% CI)	P-value
Patient age, by decade	1.07 (1.03 – 1.12)	0.001	1.10 (1.06 – 1.14)	<0.0001
Patient gender				
Men	Reference		Reference	
Women	0.81 (0.7 – 0.93)	0.003	0.86 (0.73 – 1.02)	0.08
Patient race and ethnicity				
White	Reference			
Asian	0.71 (0.60 – 0.83)	<0.0001	0.70 (0.58 – 0.84)	0.0001
Black	0.87 (0.62 – 1.22)	0.43	0.66 (0.45 – 0.96)	0.03
Hispanic	0.95 (0.74 – 1.21)	0.67	0.68 (0.55 – 0.83)	0.0001
Emergency department zones				
Fast track	Reference		Reference	
Emergent	1.18 (0.87 – 1.59)	0.29	0.97 (0.67 – 1.40)	0.87
Urgent	0.71 (0.54 – 0.93)	0.01	0.69 (0.50 – 0.95)	0.02
Vertical	0.71 (0.53 – 0.95)	0.02	0.76 (0.54 – 1.06)	0.1
Physician age, by decade	1.02 (0.91 – 1.15)	0.69	0.99 (0.86 – 1.14)	0.9
Physician gender				
Men	Reference		Reference	
Women	1.07 (0.9 – 1.27)	0.45	1.03 (0.83 – 1.29)	0.76
Physician race/ethnicity				
White	Reference		Reference	
Asian	1.05 (0.87 – 1.27)	0.62	0.89 (0.71 – 1.13)	0.34
Physician years at institution	1.01 (0.99 – 1.03)	0.14	1.01 (0.99 – 1.02)	0.44

*OR*, odds ratio; *CI*, confidence interval.
